# Additively manufactured biodegradable porous FeMn-akermanite scaffolds for critical-size bone defects: the first *in vivo* evaluation

**DOI:** 10.1016/j.mtbio.2025.102123

**Published:** 2025-07-21

**Authors:** Niko E. Putra, Jietao Xu, Marius A. Leeflang, Nicole Kops, Maria Klimopoulou, Vahid Moosabeiki, Lidy E. Fratila-Apachitei, Jie Zhou, Gerjo J.V.M. van Osch, Eric Farrell, Amir A. Zadpoor

**Affiliations:** a Department of Biomechanical Engineering, Faculty of Mechanical Engineering, Delft University of Technology, Mekelweg 2, 2628 CD, Delft, the Netherlands; b Department of Orthopedics, Zhejiang Provincial People's Hospital, Hangzhou Medical College People's Hospital, Hangzhou, Zhejiang, China; c Department of Orthopedics and Sports Medicine, Erasmus MC, University Medical Center Rotterdam, 3015 CN, Rotterdam, the Netherlands; d Department of Otorhinolaryngology, Head and Neck Surgery, Erasmus MC, University Medical Center Rotterdam, 3015 CN, Rotterdam, the Netherlands; e Department of Oral and Maxillofacial Surgery, Erasmus MC, University Medical Center Rotterdam, 3015 CN, Rotterdam, the Netherlands

**Keywords:** Biodegradable materials, Implants, Bone tissue repair, Tissue engineering, Regenerative medicine, Additive manufacturing

## Abstract

Additively manufactured (AM) iron (Fe)-based scaffolds have been developed as promising biodegradable bone-substituting biomaterials. Multi-material extrusion-based 3D printing has recently yielded Fe-manganese (Mn) alloy-based scaffolds that can resolve ferromagnetism and cytotoxicity associated with Fe-based biomaterials. Herein, we, for the first time, present the findings from *in vivo* study on extrusion-based AM FeMn-akermanite (Ak) scaffolds for critical-size bone defect repair. The scaffolds comprised Fe, 35 wt% Mn, and 20 or 30 vol% Ak, with microporous struts and 61–63 % porosity. Both scaffolds exhibited mechanical properties within the range of trabecular bone and provided suitable sites for Ca/P deposition during *in vitro* biodegradation. *In vitro* cell cultures demonstrated favorable cell responses without negating the osteogenic potential of cells. An *in vivo* study was conducted in a murine semi-orthotopic subcutaneous model. With this model, 4 bovine bone plugs were implanted subcutaneously with critical-size defects created at their cores. Scaffolds were placed into these critical-size defects to assess biodegradation and bone formation. After 16 weeks, the volume of scaffolds decreased by 6–8 %. The FeMn-20Ak scaffolds retained their yield strength and elastic modulus during the 16 weeks *in vivo*, whereas the mechanical integrity of FeMn-30Ak scaffolds deteriorated after mechanical push-out tests. Excellent osseointegration of both scaffold groups was apparent. 3D reconstruction of CT images revealed that FeMn-30Ak scaffolds had more newly formed tissue in the macro-pores than FeMn-20Ak. Altogether, our findings demonstrate the potential of AM FeMn-Ak scaffolds as biodegradable bone substitutes, encouraging further *in vivo* research in a large animal model.

## Graphical abstract

**Figure:**
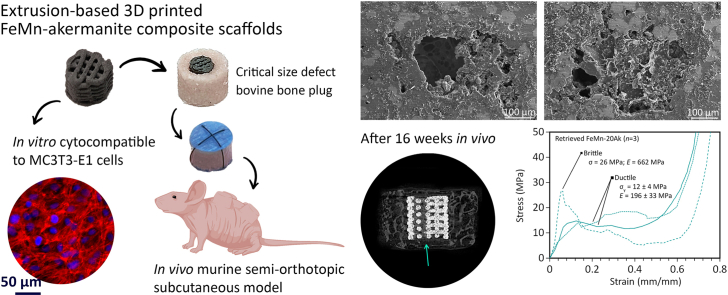

## 1 Introduction

Treating critical-size bone defects remains challenging partially due to the unavailability of ideal bone substitutes [1]. A variety of bone-substituting biomaterials have been developed in recent years. These biomaterials should be designed to fit the anatomical morphology of the bone defect and have mechanical properties similar to those of the surrounding tissue to support the healing bone and avoid stress shielding [2,3]. They should be able to biodegrade with time while maintaining sufficient mechanical integrity during bone healing [4,5]. They typically feature highly interconnected porosity to facilitate the ingrowth of new bony tissue and transport nutrients essential for bone repair [6].

Iron (Fe) is a potentially helpful biodegradable metal for biomedical applications [7]. Significant progress has been made in developing Fe-based biomaterials as biodegradable bone substitutes through *in vitro* studies. The slow biodegradation rate of Fe has been addressed by introducing porosity [8] and alloying elements, such as manganese (Mn) and silver (Ag), to form microstructural phases of different nobility, triggering local corrosion through micro-galvanic interactions [9]. The inherent ferromagnetism of Fe can be altered by alloying Fe with >28 wt% Mn to create paramagnetic FeMn alloys that are magnetic resonance (MR)-friendly [10]. Moreover, the cytocompatibility and bioactivity of Fe have been improved by incorporating bioactive ceramics into the Fe matrix. Previous studies have reported the biocompatibility of traditionally manufactured Fe-based biomaterials, such as Fe and FeMn alloys [[11], [12], [13]], Fe-Fe_3_P composites [14], and FeMnCu alloys for bone implants [15]. While these Fe-based biomaterials have demonstrated *in vivo* biocompatibility for the intended bone substitutions, they were fabricated using traditional techniques that often fail to meet the requirements of bone scaffolds for critical-size defects, such as the inability to precisely control geometry and porosity or tailor biomaterial compositions to achieve the required implant properties.

Additive manufacturing (AM) has emerged as a promising technology that offers multiple capabilities needed for fabricating porous biomaterials – ideal for bone regeneration [16]. AM has been employed to fabricate geometrically ordered porous pure Fe scaffolds [17,18] and applied to *in situ* multi-material fabrication [[19], [20], [21]]. AM of porous Fe with Mn and bioceramics allows the creation of multi-functional FeMn-based composite scaffolds that have improved biodegradation rates while being MR-friendly and cytocompatible [[22], [23], [24]]. Even though AM has been demonstrated to be viable in addressing the challenges of Fe-based bone substitutes *in vitro*, *in vivo* studies on AM Fe-based bone substitutes are scarce [[25], [26], [27]]. This knowledge gap is particularly concerning given the widely acknowledged poor correlation between the results obtained from *in vitro* and *in vivo* assessments of biodegradable metallic biomaterials.

This study presents the first *in vivo* evaluation of AM porous Fe-based scaffolds comprised of three materials – Fe, Mn, and akermanite (Ak) bioceramic – *in situ* fabricated using an extrusion-based AM technique. Based on the findings of our previous *in vitro* studies [[22], [23], [24]], we selected two promising composite materials with Fe as the base material, supplemented with 35 wt% Mn and 20 or 30 vol% Ak. One of the earlier studies demonstrated that the AM porous Fe35Mn alloy scaffolds exhibited an *in vitro* biodegradation rate (*i.e.*, 0.23 mm/year), being favorable for bone implants, as well as a paramagnetic behavior, making them compatible with magnetic resonance (MR) imaging. It was however found to be cytotoxic [22]. In a follow-up study, we addressed the cytotoxicity issue by introducing the Ak bioceramic into the Fe35Mn alloy scaffolds, which provided bioactive sites on the scaffold surface and led to the release of calcium (Ca), magnesium (Mg), and silicon (Si) ion release [23]. The selection of Ak as the bioceramic of choice was based on findings concerning its mechanical properties and dissolution rate, which are superior to those of other bioceramics, such as CaSiO_3_ [28]. We demonstrated that the AM porous Fe35Mn scaffolds with 20 vol% or 30 vol% Ak had enhanced *in vitro* biodegradation rates (*i.e.*, 0.24 mm/year or 0.27 mm/year, respectively), while being MR-friendly, cytocompatible, and osteogenic [24]. In addition, the scaffolds with 20 vol% Ak exhibited a lower biodegradation rate but offered better mechanical properties than those with 30 vol% Ak [24]. Here, we report the findings from the *in vivo* evaluation of the extrusion-based AM porous FeMn-Ak scaffolds using a murine semi-orthotopic bone defect model [29]. The scaffolds were implanted into a cylindrically shaped bovine trabecular bone with a critical-size defect and implanted subcutaneously in nude mice for 16 weeks along with longitudinal micro-computed tomography (μCT) evaluation. After retrieval, we assessed the retrieved scaffolds’ *in vivo* biodegradation behavior, mechanical properties, osseointegration, and possible local and remote adverse tissue effects.

## 2 Materials and methods

### 2.1 Preparation of FeMn-Ak ink and 3D printing

Fe elemental powder (purity = 99.88 wt%; particle sizes <63 μm; spherical morphology; α-phase [22]) and Mn elemental powder (purity = 99.86 wt%; particle sizes <45 μm; irregular morphology; α-phase [22]) were obtained from Material Technology Innovations Co. Ltd. (China). The Ak powder (Ca_2_MgSi_2_O_7_; particle sizes <45 μm; irregular morphology, Shanghai Institute of Ceramics, Chinese Academy of Sciences) was synthesized from tetraethyl orthosilicate [(C_2_H_5_O)_4_Si, TEOS], magnesium nitrate hexahydrate [Mg(NO_3_)_2_·6H_2_O], and calcium nitrate tetrahydrate [Ca(NO_3_)_2_·4H_2_O] using a sol-gel technique and calcination, as reported in Ref. [30].

FeMn-Ak powder mixtures were prepared by blending Fe, 35 wt% Mn, and either 20 or 30 vol% Ak powders, hereafter referred to as FeMn-20Ak and FeMn-30Ak, respectively. The inks were created by mixing the powder mixtures with a binder containing 5 wt% hydroxypropyl methylcellulose (Mw ∼86 kDa, Sigma Aldrich, Germany) in a hydro-alcoholic solvent [14]. The FeMn-Ak powder mixture in the ink conformed to a volume ratio of 47.45 %. Then, the inks were used to fabricate porous scaffolds employing a 3D BioScaffolder 3.2 printer (GeSiM Bioinstruments and Microfluidics, Germany).

The scaffolds were designed in a lay-down pattern with 0° and 90° switching every other layer to construct cylindrical porous specimens (*ϕ* = 4.0 mm and *h* = 4.0 mm). The scaffolds had a strut size and spacing of 410 μm and 400 μm, respectively. 3D printing was performed at printing pressures of 325 kPa and 360 kPa for the FeMn-20Ak and FeMn-30Ak inks, respectively, at a printing speed of 3.5 mm/s. Afterward, the green scaffolds were placed into a tube furnace (STF16/180, Carbolite Gero Ltd., UK) under high-purity flowing argon (grade 6.0) for heat treatment. Debinding was performed at 350 °C for 3 h and sintering at 1200 °C for 6 h. The specimens were cooled naturally to room temperature. The as-sintered specimens were ultrasonically cleaned in isopropyl alcohol for 15 min for further tests. The specimens were sterilized in a dry oven at 120 °C for 2 h for *in vitro* and *in vivo* experiments.

### 2.2 Characterization of the FeMn-Ak scaffolds

The morphologies and chemical compositions of the porous FeMn-Ak scaffolds were examined using a scanning electron microscope (SEM, JEOL JSM-IT100, Japan) equipped with an energy dispersive X-ray spectroscope (EDS, JEOL JSM-IT100, Japan), and μCT scan (Quantum GX, Perkin Elmer, USA) and the μCT images were analyzed using the Dragonfly software (version 2022.1.0.1249, Object Research Systems, Canada)

. The absolute porosity value of the scaffolds was determined using Equation (1):

(1) $$\varphi_{a}=\left(1-\frac{m/\rho_{FeMn-Ak}}{V_{bulk}}\right)\times 100\%$$,

where *φ*_*a*_ is the absolute porosity [%], *m* is the mass [g] of the as-sintered scaffold, *V*_*bulk*_ is the bulk volume [cm^3^], and *ρ*_*FeMn-Ak*_ is the theoretical density of the FeMn-Ak composite (*i.e.*, 6.68 g/cm^3^ for FeMn-20Ak and 6.22 g/cm^3^ for FeMn-30Ak).

### 2.3 *In vitro* immersion tests

To evaluate the *in vitro* biodegradation behavior, the sterilized FeMn-20Ak and FeMn-30Ak scaffolds (*ϕ* = 4.0 mm and *h* = 4.0 mm) were immersed in the revised simulated body fluid (r-SBF [31]) for 4, 6, and 8 weeks (in triplicate, for each time point) under static conditions, 37 ± 0.5 °C, 95 % relative humidity, and 5 % CO_2_ atmosphere. 6.7 mL of r-SBF per 1 cm^2^ scaffold surface area was used [32]. The value of the surface area of the specimen was based on the initial scaffold design value. The r-SBF medium filtered using a 0.22 μm pore size filter (Merck Millipore, Germany) was renewed every 2 weeks. The r-SBF was collected every 2 weeks, and the concentrations of soluble ions (*i.e.*, Fe, Ca, Mg, Si, and PO_4_) were measured using an inductively coupled plasma-optical emission spectrometer (ICP-OES, iCAP 6500 Duo, Thermo Scientific, USA).

### 2.4 *In vitro* cell tests

#### 2.4.1 Effect of scaffold extracts on cell viability

We evaluated the effects of scaffold extracts on the morphology and number of cells. First, the FeMn-20Ak and FeMn-30Ak scaffold extracts were prepared by incubating the sterile scaffolds in the cell culture medium (containing α-minimum essential medium (α-MEM) supplemented with 10 % fetal bovine serum (FBS) and 1 % penicillin/streptomycin (p/s)) at an extraction ratio of 5 cm^2^/mL. The extractions were performed in a cell culture incubator for 72 h [33]. The FeMn-20Ak and FeMn-30Ak extract media were stored at 4 °C for 24 h prior to use.

Preosteoblasts (MC3T3-E1, Sigma Aldrich, Germany, 5 × 10^3^ cells per cm^2^, in a 48-well plate) were cultured for a day in 200 μL cell culture medium and then in cell culture medium with 10 % (v/v) of the scaffold extract media, following the recommendations for the cytotoxicity testing of biodegradable metals [34], for 3 days. Cells cultivated in a fresh cell culture medium were used as the control group.

After culture, the cells were washed with PBS (Thermo Fisher Scientific, USA), fixed using 4 % formaldehyde (Sigma Aldrich, Germany) at room temperature for 15 min, then washed with PBS and permeabilized with 0.5 % triton/PBS (Sigma Aldrich, Germany) at 4 °C for 5 min. Then, 1 % bovine serum albumin/PBS (BSA, Sigma Aldrich, Germany) was added per well and incubated for 5 min. Consecutively, 1:1000 rhodamine phalloidin (Thermo Fisher Scientific, USA) in 200 μL 1 % BSA/PBS was added per well, followed by 1 h incubation at 37 °C. Afterward, the specimens were rinsed in 0.5 % tween/PBS (Sigma Aldrich, Germany) and washed with PBS. Then, a 1:200 4′, 6-diamidino-2-phenylindole (DAPI, Life Technologies, USA) in 200 μL PBS was added, and the specimens were incubated for 3 min. Finally, the specimens were washed, and the F-actin and nuclei were observed using a fluorescence microscope (ZOE cell imager, Bio-Rad, USA). First, the DAPI images were converted to 8-bit grayscale to count the number of cells. Cells were identified using manual thresholding and applying a watershed function to separate cell clusters. Cells were counted automatically with ImageJ's analyzed particle function (NIH, USA).

#### 2.4.2 Osteogenic gene expression analysis

To assess whether the biodegradable scaffolds could affect cells in the adjacent bone, we cultured preosteoblasts in the presence of the FeMn-20Ak and FeMn-30Ak scaffold extracts and evaluated the effect on the osteogenic activity of cells. Preosteoblasts (5 × 10^3^ cells per cm^2^ in 48-well plate, in triplicate for each time point) were cultured in 200 μL osteogenic medium (consisting of α-MEM, supplemented with 10 % FBS, 1 % p/s, 1:1000 ascorbic acid, and 1:500 β-glycerophosphate) that contained the 10 % of FeMn-20Ak and FeMn-30Ak scaffold extract media for 7, 14, and 21 days. The media were refreshed every 2–3 days. The cells cultivated in the osteogenic medium without extracts were used as the control group.

Real-time-polymerase chain reaction (RT-PCR) analysis was performed to quantify the expression of genes associated with osteogenesis, such as alkaline phosphatase (*Alpl*), osteopontin (*Opn*), and osteocalcin (*Bglap*). Ubiquintin C (*Ubc*) was selected as the reference gene. At the designated time point, ribonucleic acid (RNA) was isolated with the RNeasy micro kit (Qiagen, Germany) and converted to complementary deoxyribonucleic acid (cDNA) using a Quantitect Reverse Transcription kit (Qiagen, Germany). RT-qPCR was conducted on the Rotor X gene PCR system and the Quantinova SYBR Green PCR kit (Qiagen, Germany), with 5 ng cDNA per reaction. The primers are listed in Table 1. The delta threshold cycle (ΔCT) values were calculated and reported.

**Table 1:** Primer sequences for RT-qPCR.

Gene	Forward primer	Reverse primer
*Alpl*	gttgccaagctgggaagaacac	cccaccccgctattccaaac
*Opn*	ctttcactccaatcgtcccta	gctctctttggaatgctcaagt
*Bglap*	gccatcaccctgtctcctaa	tgtaggcggtcttcaagccat
*Ubc*	agcccagtgttaccaccaag	acccaagaacaagcacaagg

#### 2.4.3 Direct cell culture on the scaffolds

To evaluate the morphology of cells on the FeMn-20Ak and FeMn-30Ak scaffolds, we performed direct cell culture of preosteoblasts (MC3T3-E1, 5 × 10^4^ cells per specimen) on the scaffolds (*ϕ* = 7.0 mm and *h* = 0.6 mm) in 6-well plates containing 8 mL of osteogenic cell culture medium (consisting of α-MEM, supplemented with 10 % FBS, 1 % p/s, 1:1000 ascorbic acid, and 1:500 β-glycerophosphate). The medium was refreshed every 2–3 days. The morphology of the cells cultured on the scaffolds for 4 and 14 days (in triplicate for each time point) was observed using SEM (JEOL JSM-IT100, Japan) with a voltage of 15 kV and a working distance of 10 mm.

### 2.5 *In vivo* bone defect model in mice

**Fig. 1:**
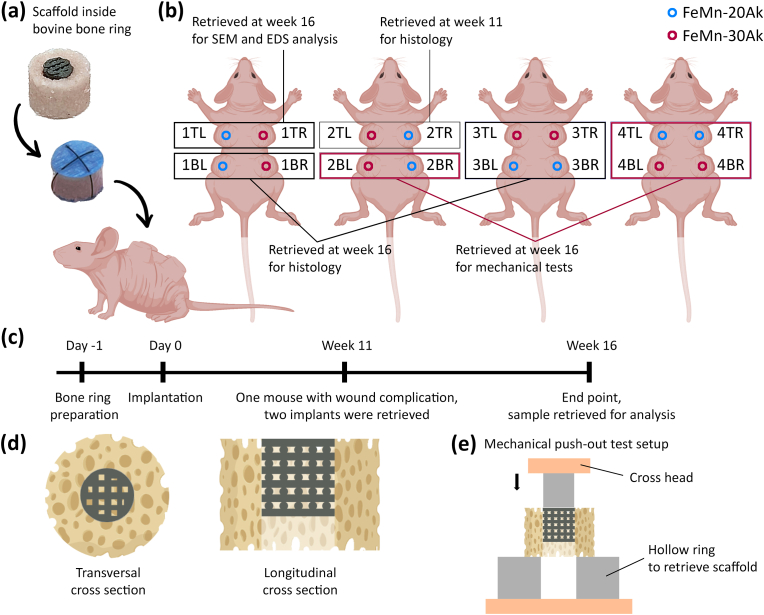
A schematic illustration of the murine semi-orthotopic bone defect model. (a) An AM porous FeMn-akermanite composite scaffold in the critical size defect in an explant of bovine trabecular bone, implanted subcutaneously in a mouse. (b) The *in vivo* experimental setup with specimen locations, labels (TL = top left, TR = top right, BL = bottom left, and BR = bottom right), and their corresponding analysis. (c) Timeline of the *in vivo* experiment. (d) Transversal and longitudinal cross-section of the construct. (e) Mechanical push-out test setup.

A semi-orthotopic murine model established in our laboratory was used to assess the scaffolds' osseointegration capacity. This model enabled us to study several critical-size bone defects in a small animal model by using a cylindrically shaped bovine trabecular bone explant with a defect in the core that can be filled with a scaffold [29]. The bone-scaffold construct is then implanted subcutaneously in mice (Fig. 1a). The animal experiments were conducted in the animal facility of Erasmus MC, University Medical Center Rotterdam, after ethical approval (under license number 101002015114 and protocol number 15-114-09). Bone cylinders (with *ϕ* = 8 mm and *h* = 5 mm) were harvested from the metacarpal-phalangeal joints of 6 to 8-month-old calves (LifeTec, The Netherlands). A critical-size bone defect (*ϕ* = 4 mm and *h* = 5 mm) was created at the center of the bone cylinder. The created bone rings were kept overnight in α-MEM supplemented with 10 % FBS, 50 μg/mL gentamycin, and 1.5 μg/mL fungizone (all from Thermo Fischer Scientific, The Netherlands). Prior to implantation, the hollow bone defects were filled with either the FeMn-20Ak scaffolds or the FeMn-30Ak scaffolds. All the bone-scaffold constructs were covered with a circular 8 mm polytetrafluoroethylene (PTFE) membrane to minimize the direct ingrowth of tissue into the bone defects. The surgery was performed on 12-week-old NMRI-Fox1nu female mice (Janvier Labs, France), and 4 mice were used in this study. The animals were randomly assigned and housed under specific pathogen-free conditions with a regular day/night light cycle. They were allowed to adapt to the animal facility environment for 7 days before surgery. Food and water were available *ad libitum*.

Each bone-scaffold construct was implanted in a separate subcutaneous pocket on the back of the mice under 2.5–3 % isoflurane anesthesia (1000 mg/g, Laboratorios Karizoo, India). Four constructs were implanted per mouse. Each mouse was implanted with two FeMn-20Ak specimens and two FeMn-30Ak specimens at designated locations (Fig. 1b). After the construct placement, the incisions were closed with staples (Fine Science Tools, Canada). Pre- and post-operative analgesia was provided through a subcutaneous injection of 0.05 mg/kg body weight of buprenorphine (Chr. Olesen & Co, Gentofte, Denmark). The mice also received a subcutaneous prophylactic amoxicillin (Dopharma, The Netherlands) injection of 25 mg/kg body weight to prevent infection.

At week 11, two constructs (one specimen per sample group) from the same mouse were retrieved under 2.5–3 % isoflurane anesthesia due to a persisting wound issue. These specimens underwent micro-computed tomography (μCT) scan and histological analysis. At week 16, the mice were sacrificed by cervical dislocation under 2.5–3 % isoflurane anesthesia, and the 14 remaining constructs were harvested. Six constructs (3 specimens per sample group) were dehydrated in 70 % ethanol, air dried, and then subjected to push-out mechanical testing. Two constructs (1 specimen per sample group) were examined morphologically and their chemical compositions were analyzed using EDS attached to SEM. Six constructs (3 specimens per sample group) were prepared for histological analysis. For histological analysis, organs (*i.e.*, brains, livers, kidneys, lungs, hearts, and spleens) were collected immediately after cervical dislocation.

### 2.6 μCT scan and image segmentation

Structural integrity and biodegradation changes were examined over time for the longitudinal assessment of the scaffolds’ osseointegration. μCT scanning (Quantum GX, Perkin Elmer, USA) was performed on the mice every two weeks at a voltage of 90 kV, a current of 88 μA, a field of view (FOV) of 36 mm, and an isotropic voxel size of 72 μm. In addition, μCT scans were performed on the retrieved construct specimens prior to fixation in 70 % ethanol at the same voltage and current values, a FOV of 18 mm, and a higher resolution of 36 μm isotropic voxel size. The scan time was 4 min. All the scans were conducted using an X-ray filter made of copper (Cu) with a thickness of 0.06 mm and aluminum (Al) with a thickness of 0.5 mm. To ensure the accuracy of those scans, the μCT was calibrated using a phantom with a known density of 0.75 g/cm^3^ before each set of scans.

The μCT scan images of the construct specimens at week 16 were analyzed using the Dragonfly software (version 2022.1.0.1249, Object Research Systems, Canada). The scaffold material and newly formed tissue were segmented using the Otsu thresholding algorithm in the software where the intensity ranges were automatically detected to differentiate the tissue (*i.e.*, 4971–9249) and scaffold material (*i.e.*, 12762–32767). Cylindrical regions of interest (ROI) were locally determined to capture the scaffolds (Fig. S1a) and the critical-size defects (Fig. S1b). To determine the biodegradation rate, the volume of the scaffolds excluding tissue ingrowth (*n* = 7 per group) after 16 weeks *in vivo* was compared to the initial volume of the scaffolds (*n* = 1 per group, determined at day 0). The ratio of tissue ingrowth in the scaffold (*n* = 7) was calculated by dividing the volume of bone tissue within the scaffold (BV_*i*_) by the total volume of the macro-porosity of the scaffolds (TV_*pore*_). Total newly formed tissue (*n* = 7) was quantified by dividing the total volume of newly formed tissue by the total volume of the critical size defect (BV/TV).

### 2.7 Cross-sectional characterizations of the bone-scaffold constructs

For SEM and EDS analysis, two construct specimens containing the FeMn-20Ak and FeMn-30Ak scaffolds after 16 weeks *in vivo* were fixated in 70 % ethanol, followed by a dehydration step in 96 % and absolute ethanol. Then, the constructs were dried overnight prior to imaging. First, the overall surface morphology of the constructs was observed using SEM, and the chemical composition of the surface was determined using EDS line analysis. Afterward, the constructs were ground with sandpaper on the transverse plane to expose the interface of the scaffold and bone ring (Fig. 1d). The morphologies of the scaffold struts, macro-pores, and the surrounding bone ring at the exposed transverse cross-section of the constructs were observed using SEM. The chemical composition of the scaffold struts with macro-pores was determined using EDS mapping analysis. The changes in chemical composition across the scaffold-to-bone interface were determined using EDS line analysis. After evaluating the transversal plane, the constructs were sectioned to expose the longitudinal plane (Fig. 1d). Similar studies were performed at the exposed longitudinal cross-sections of the constructs.

### 2.8 Mechanical tests

To evaluate the integration of the scaffold in the surrounding bone, the specimens dedicated for mechanical tests (in triplicate per sample group) were placed in a custom-built setup for push-out tests (Fig. 1e). The setup utilized a pin (with *ϕ* = 3.8 mm and *h* = 15 mm) to push out the scaffolds using a universal mechanical testing machine (Lloyd Instrument LR5K, UK) at 3 mm/min. Bone-scaffold constructs (in triplicate) that were not implanted were used as controls. The force and displacement curves were used to determine each specimen's peak force (*F*_*peak*_) value. The shear area (*A*_*shear*_) was determined from the lateral surface area where the specimens made direct contact with the surrounding material during displacement (=π × *ϕ* × *h*, where *ϕ* is the diameter of the implant and *h* is the displacement measured during the push-out test). The shear strength (*τ*) value was calculated by dividing *F*_*peak*_ by *A*_*shear*_. Furthermore, the compressive mechanical properties of the porous FeMn-Ak scaffolds (both the as-sintered and as-retrieved specimens after the push-out tests) were evaluated using a universal mechanical testing machine (Lloyd Instrument LR5K, UK) at a crosshead speed of 3 mm/min. The compressive 0.2 % offset stress (referred to as the yield strength) and the stiffness of the porous FeMn-Ak scaffolds were determined according to ISO 13314:2011 [35]. All the tests were performed in triplicate.

### 2.9 Histology analysis

After the μCT scan, the retrieved construct specimens dedicated for histology analysis were fixed in 70 % ethanol for 7 days. After dehydration in 96 % and 100 % ethanol, the specimens were embedded in methyl methacrylate (MMA). The embedded specimens were trimmed using a butcher saw (Bizerba) and glued onto holders to fit into annular saws (Leica SP 1600 saw microtome, Leica AG). Four to five serial sections were produced (ranging in thickness from 320 to 490 μm) from each specimen. Contact radiographs of the sections were made using a Faxitron X-Ray Cabinet (Faxitron X-Ray Corporation) before proceeding. The most central section of the defect was chosen for further processing. The sections were glued onto opaque Plexiglas slides, ground, and polished (Exakt Micro Grinding System, Exakt Apparatebau) using varying roughnesses of grinding papers (P1200–P4000, Struers GmbH) to obtain sections with a final thickness of approximately 210–230 μm.

To stain the sections, the slides were first etched using 1 % formic acid, stained with a heated solution of 15 % Giemsa (Sigma Aldrich, Germany), rinsed, counterstained with a solution of 1 % Eosin Y (Sigma Aldrich, Germany), differentiated in 70 % ethanol, and then dehydrated. The stained sections were imaged using bright field illumination with an upright microscope (Olympus BX63, Japan) and image acquisition software (cellSens V4.1, Olympus, Japan) using tile imaging with a 20 × objective. The histological images were blindly evaluated for the interactions of bone and scaffold at the interface, bone ingrowth in the center pores, vascularization, and signs of inflammation and necrosis. In addition, organs harvested from mice were fixed in formalin for 7 days, embedded in paraffin, and sectioned at 6 μm. Then, the specimens were dewaxed, and a Hematoxylin and Eosin Y (H&E, Merck, US) staining was performed. In addition, a Perls' Prussian blue iron staining (Clin-Tech, UK) with a nuclear fast red counterstaining (Sigma-Aldrich, Germany) was performed on the liver specimens.

### 2.10 Statistical analysis

The results are presented as mean values with standard deviations. The datasets were tested for normality using the Shapiro-Wilk test. The BV_i_/TV_pore_ results (*n* = 7) were analyzed using an unpaired *t*-test. The statistical analysis was performed using GraphPad Prism (GraphPad Software, USA).

## 3 Results

### 3.1 Characteristics, mechanical properties, and *in vitro* biodegradation behavior of the scaffolds

**Fig. 2:**
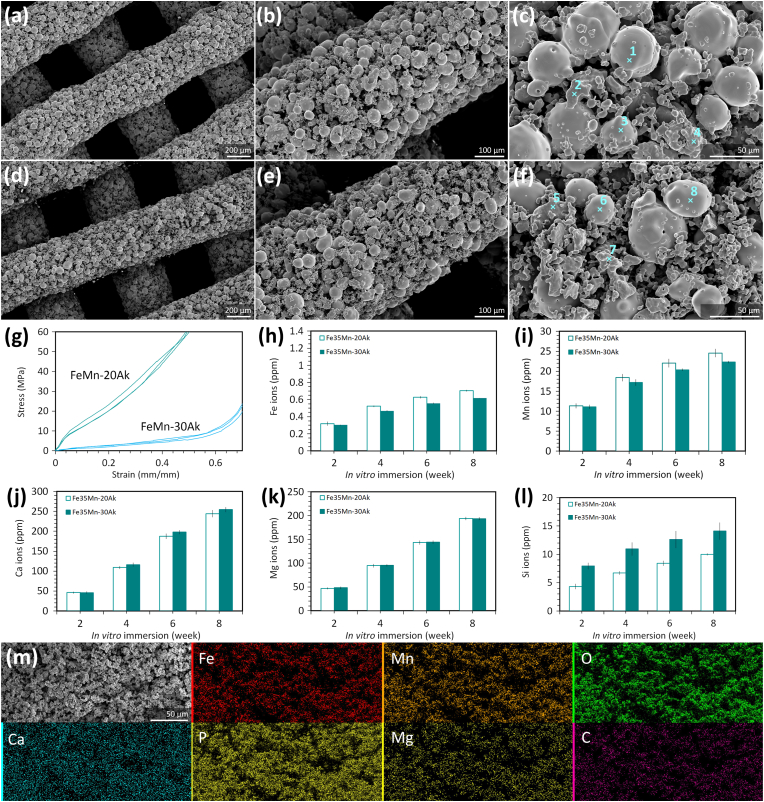
Characteristics, mechanical properties, and *in vitro* biodegradation behavior of the FeMn-akermanite composite scaffolds. SEM images of (a–c) the FeMn-20Ak scaffolds and (d–f) the FeMn-30Ak scaffolds, showing (a, d) the as-sintered macro-porous structure, (b, e) the struts, and (c, f) points on the sintered powder particles in the struts for EDS analysis. (g) The compressive stress-strain curves of the scaffolds (*n* = 3 for each group). The concentrations of (h) Fe, (i) Mn, (j) Ca, (k) Mg, and (l) Si ions in the r-SBF medium over time. (m) The SEM image and EDS chemical mapping of the biodegradation products on the surface of the FeMn-20Ak scaffolds after 8 weeks of *in vitro* immersion.

The extrusion-based AM porous FeMn-20Ak scaffolds (Fig. 2a, 2b, and 2c) had an average strut diameter of 423 ± 2 μm measured in SEM (429 ± 6 μm by μCT), and an interconnected porous structure with a pore spacing between the struts having an average size of 385 ± 3 μm measured in SEM (381 ± 4 μm by μCT). The morphology of the FeMn-30Ak scaffolds was similar (Fig. 2d, 2e, and 2f), with a strut diameter of 426 ± 4 μm measured in SEM (432 ± 5 μm by μCT) and a pore spacing of 382 ± 5 μm measured in SEM (378 ± 3 μm by μCT). The absolute porosity of FeMn-20Ak and FeMn-30Ak scaffolds were 61 ± 2 % and 63 ± 2 %. The struts of the scaffolds comprised sintered spherically shaped particles containing Fe and Mn and irregularly shaped particles containing the elements of Ak (*i.e.*, Ca, Mg, Si, and O) together with penetrated Fe and Mn elements (Table 2). Both scaffold groups could be visually distinguished by comparing the chemical compositions of the sintered particles in the struts. The FeMn-20Ak scaffolds contained more spherically shaped FeMn alloy particles (Fig. 2b–e), while the FeMn-30Ak scaffolds had a higher proportion of irregularly shaped Ak particles (Fig. 2c–f). Under uniaxial compression (Fig. 2g), the AM porous FeMn-20Ak scaffolds had an average yield strength of 11 ± 2 MPa and an elastic modulus of 174 ± 32 MPa. The compressive mechanical properties of the AM porous FeMn-30Ak scaffolds were much lower, with a yield strength of 1.2 ± 0.3 MPa and an elastic modulus of 30 ± 4 MPa.

**Table 2:** Chemical compositions (wt%) of the sintered powder particles in the scaffold struts, determined by EDS analysis at points indicated in Fig. 2c and f.

Scaffold	EDS point	Fe	Mn	Ca	Mg	Si	O
**FeMn-20Ak**	**1**	69.40	30.60	–	–	–	–
	**2**	3.33	25.88	27.86	7.16	18.84	16.94
	**3**	69.61	30.39	–	–	–	–
	**4**	4.28	19.64	25.43	7.59	18.07	25
**FeMn-30Ak**	**5**	1.64	20.40	30.51	8.73	19.05	19.68
	**6**	70.50	29.50	–	–	–	–
	**7**	9.39	36.42	41.92	1.14	6.81	4.32
	**8**	71.22	28.78	–	–	–	–

The *in vitro* biodegradation behavior of the AM porous FeMn-Ak scaffolds was evaluated in the r-SBF medium with the same ion concentrations as the human blood plasma (Table S1). During the *in vitro* immersion tests, the concentrations of Fe, Mn, Ca, Mg, and Si ions in the medium steadily increased with time. The concentrations of Fe ions released from the FeMn-20Ak and FeMn-30Ak scaffolds into the medium were comparable (*i.e.*, 0.6–0.7 ppm after 8 weeks of immersion) (Fig. 2h). By the end of the 8^th^ week, the FeMn-20Ak scaffolds released a total of 25 ± 1 ppm Mn ions, slightly more than that from the FeMn-30Ak scaffolds (*i.e.*, 22.3 ± 0.3 ppm) (Fig. 2i). The higher volume fraction of Ak in the FeMn-30Ak scaffolds resulted in greater concentrations of Ca (Fig. 2j) and (Fig. 2l) Si ions released into the r-SBF compared to those from the FeMn-20Ak scaffolds. However, the concentrations of Mg ions were comparable (Fig. 2k). In addition to ion release, these elements precipitated into the biodegradation products, covering the surfaces of the FeMn-Ak scaffolds. The biodegradation products exhibited a particulate, dense morphology (Fig. S2a–b), which could prevent oxygen from diffusion, thereby hindering further biodegradation of the scaffolds. Based on the EDS mapping, the biodegradation products contained Fe, Mn, O, Ca, P, Mg, and C (Fig. 2m–S2). The Ca/P ratio in the biodegradation products on the FeMn-20Ak scaffolds was 6.6, while that on the FeMn-30Ak scaffolds was 2.03. This suggests that the FeMn-30Ak scaffolds attracted relatively more phosphate deposition from the r-SBF on their surface, indicating a greater potential for apatite formation.

### 3.2 *In vitro* cell response and osteogenic potential of the scaffolds

**Fig. 3:**
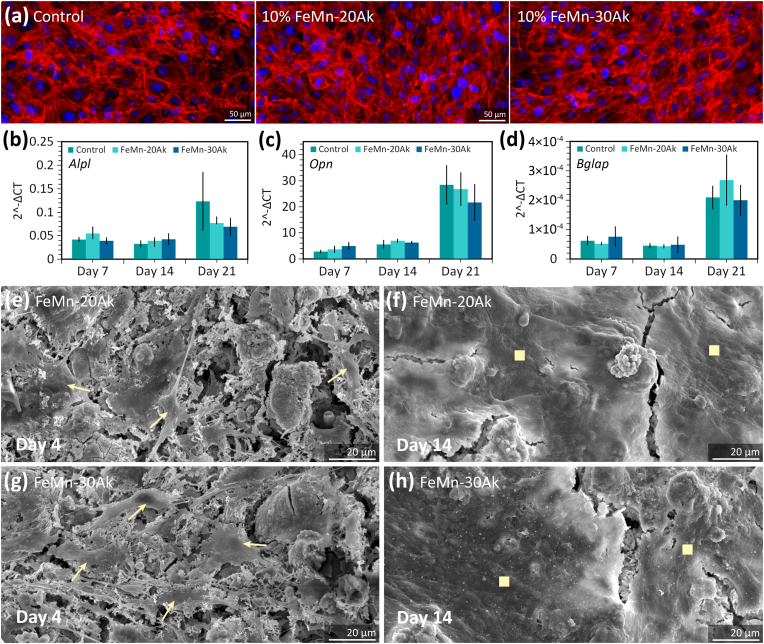
*In vitro* cell responses and the effects of the extracts on the osteogenic potential of cells. The representative images of (a) rhodamine phalloidin (red) and DAPI (blue) fluorescence staining of preosteoblasts after 3 days of culture in standard cell culture media (control group) and 10 % FeMn-akermanite extracts. The 2^−ΔCT^ values of (b) *Alpl*, (c) *Opn*, and (d) *Bglap* genes after 7, 14, and 21 days of cell culture in osteogenic cell culture media with 10 % FeMn-akermanite extracts and without extracts (control). The bars represent the mean values, while the error bars indicate the standard deviation (*n* = 3 for each time point). The morphologies of the cells on the struts of (e, f) FeMn-20Ak and (g, h) FeMn-30Ak composite scaffolds, as visualized by SEM on day 4 (the arrows point cells) and day 14 (square symbols indicate cell extracellular matrix layer).

The effect of the FeMn-Ak extract concentration on the MC3T3-E1 preosteoblasts cell count was evaluated. Cell counts in the 10 % FeMn-20Ak scaffold extract medium (*i.e.*, 887 ± 94 cells per mm^2^) or the 10 % FeMn-30Ak scaffold extract medium (*i.e.*, 766 ± 53 cells per mm^2^) were comparable to the control group (*i.e.*, 715 ± 154 cells per mm^2^) after 3 days of culture. The cells cultured with the 10 % FeMn-Ak extracts displayed a cytoskeleton morphology similar to the control group on the culture plate (Fig. 3a). Moreover, to assess whether the presence of the biodegradable scaffold could affect the osteogenic capacity of the cells in the adjacent bone, we cultured preosteoblasts in the presence of the 10 % FeMn-20Ak and FeMn-30Ak scaffold extracts for longer times and assessed osteogenic genes expression. The FeMn-Ak scaffold extracts did not negatively affect the expression of *Alpl*, *Opn*, and *Bglap* genes associated with the osteogenic potential of cells (Fig. 3b, 3c, 3d).

The cell response on the FeMn-Ak scaffolds was evaluated by directly cultured cells. Cells were present on the FeMn-20Ak and FeMn-30Ak scaffolds with a spread morphology, covering the surface of the struts, as observed on the specimens after 4 days of culture (Fig. 3e and 3g). Over time, the cell layer became denser, indicating that cells proliferated and produced an extracellular matrix on the scaffold surface (Fig. 3f and 3h). Altogether, the *in vitro* performance of the AM porous FeMn-Ak scaffolds was highly encouraging, warranting the next step of *in vivo* evaluation.

### 3.3 *In vivo* performance of the FeMn-Ak scaffolds for bone defect repair

#### 3.3.1 Clinical observations and biosafety

Over 16 weeks, all mice remained in good health, with no signs of distress or behavioral abnormalities. No lesions or dermatological issues were observed, except for one mouse that developed wound complications at two implantation sites. The affected mouse underwent a minor surgical procedure under anesthesia to remove two implants, after which the wounds were closed with staples. The mouse recovered well, and no further complications were observed. The organs were collected at the end of the *in vivo* study. We observed no major pathological changes in the H&E stained sections of heart, liver, spleen, lungs, kidneys, and brain (Fig. S3a–f). In addition, we performed Perl's Prussian blue staining on the organs and detected Fe or Mn deposit exclusively in the liver (Fig. S3g–i). We observed an early stage Fe or Mn accumulation within Kupffer cells, characteristic of hemosiderosis or manganism, without signs of hepatic damage or progression to hemochromatosis. Occasional multinuclear hepatocytes were observed, a common and non-pathological feature in rodent livers.

#### 3.3.2 In vivo biodegradation behavior of the FeMn-Ak scaffolds

**Fig. 4:**
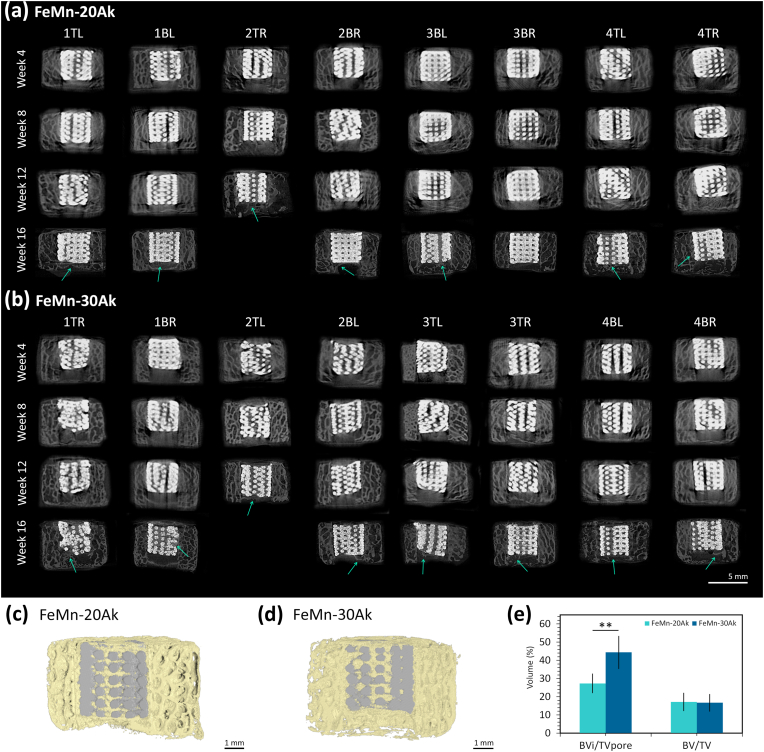
Longitudinal μCT images of the bone-scaffold constructs: (a) FeMn-20Ak and (b) FeMn-30Ak with a voxel resolution of 72 μm at weeks 4, 8, and 12, providing general overviews of the scaffolds in the constructs. At week 16, the μCT scan with a higher voxel resolution of 36 μm shows newly formed tissue in the critical-size defect, indicated by the green arrow. The μCT image reconstructions of (c) the FeMn-20Ak and (d) FeMn-30Ak bone-scaffold constructs after 16 weeks of implantation. (e) The *BV*_*i*_*/TV*_*pore*_ and *BV/TV* values at week 16. The bars represent the mean values, and the error bars indicate the standard deviations (*n* = 7, unpaired *t*-test, ∗∗ = *p* < 0.01).

During the *in vivo* experiments, the bone-scaffold constructs in mice were monitored using μCT to observe their biodegradation over time. No apparent volume changes were observed in the scaffolds (Fig. 4). After retrieval, a higher resolution μCT scan and 3D reconstruction of the construct specimens were performed. Compared to the initial scaffold volume, the AM FeMn-20Ak scaffolds had an average volume loss of 5 ± 2 %, lower than the FeMn-30Ak scaffolds, which had an average volume loss of 8 ± 3 %.

**Fig. 5:**
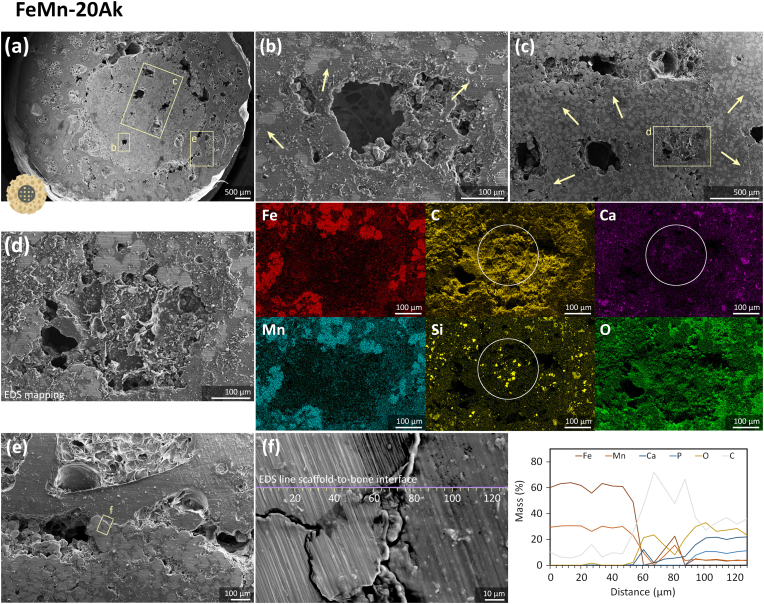
(a) The transverse cross-section of the FeMn-20Ak bone-scaffold construct showing (b, c) the macro-pores and the micro-pores penetrated by biodegradation products (in the dark gray color, as pointed by the arrows). (d) The macro-pores are filled with tissue-like structure and EDS mapping, with the circles indicating Ca and Si together with a tissue-like presence of C in the macro-pores. (e) Bone-to-scaffold interface, and (f) EDS line analysis across the bone-scaffold interface.

**Fig. 6:**
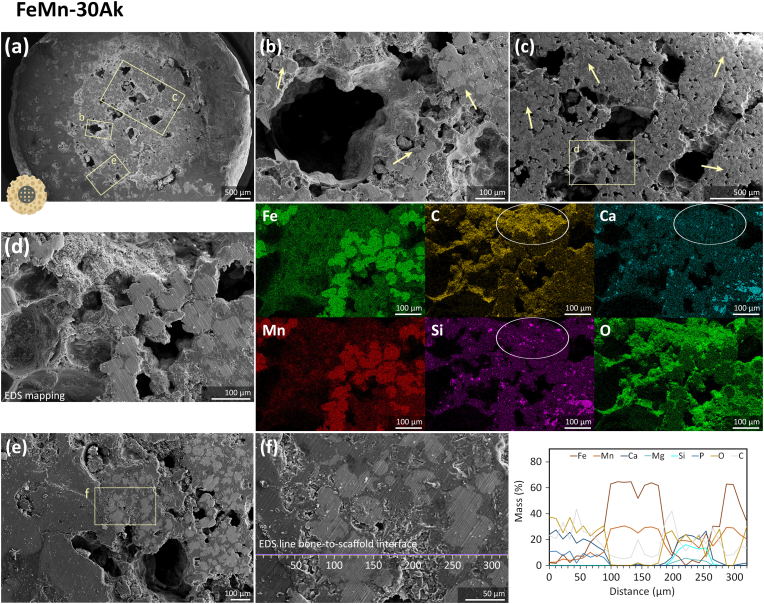
(a) The transverse cross-section of the FeMn-30Ak bone-scaffold construct, showing (b, c) the macro-pores and the micro-pores penetrated by the biodegradation products (in the dark gray color, as pointed by the arrows). (d) The struts with tissue-like coverage and EDS elemental mapping at the strut site, with the circles indicating Ca and Si together with a tissue-like presence of C on the strut surface. (e) Bone-to-scaffold interface and (f) EDS line analysis across the bone-scaffold interface.

Next, we examined the constructs' cross-section to observe the scaffold struts' morphology after the *in vivo* tests. The geometrical porous structure of the FeMn-20Ak and FeMn-30Ak scaffolds was visible after 16 weeks of implantation, indicating that the extent of *in vivo* biodegradation was limited (Fig. 4, Fig. 5, Fig. 6a). Although the biodegradation rates of both scaffolds were comparable, the struts of the FeMn-20Ak scaffolds appeared to be more compact (Fig. 5b and c) than those of the FeMn-30Ak scaffolds (Fig. 6b and c). Biodegradation was accompanied by the diffusion of Fe and Mn present in the biodegradation products into the neighboring bone ring at the interfaces (Figs. S4b and S5b). The examination of the cross-section of the FeMn-20Ak (Fig. 5b and 5c) and FeMn-30Ak (Fig. 6b and 6c) constructs revealed that dense biodegradation products (in the dark gray color, as pointed by the arrows) had formed and penetrated the spherical powder particles of the scaffold struts. The dense biodegradation products comprised a mixture of Fe, Mn, Ca, Si, C, and O, as identified by EDS mapping (Fig. 5, Fig. 6d). Histological images of both FeMn-Ak scaffolds at different magnifications showed the diffusion of the scaffold's biodegradation products into the surrounding bone tissue interfaces (Fig. 8).

#### 3.3.3 Osseointegration and mechanical properties of the FeMn-Ak scaffolds

**Fig. 7:**
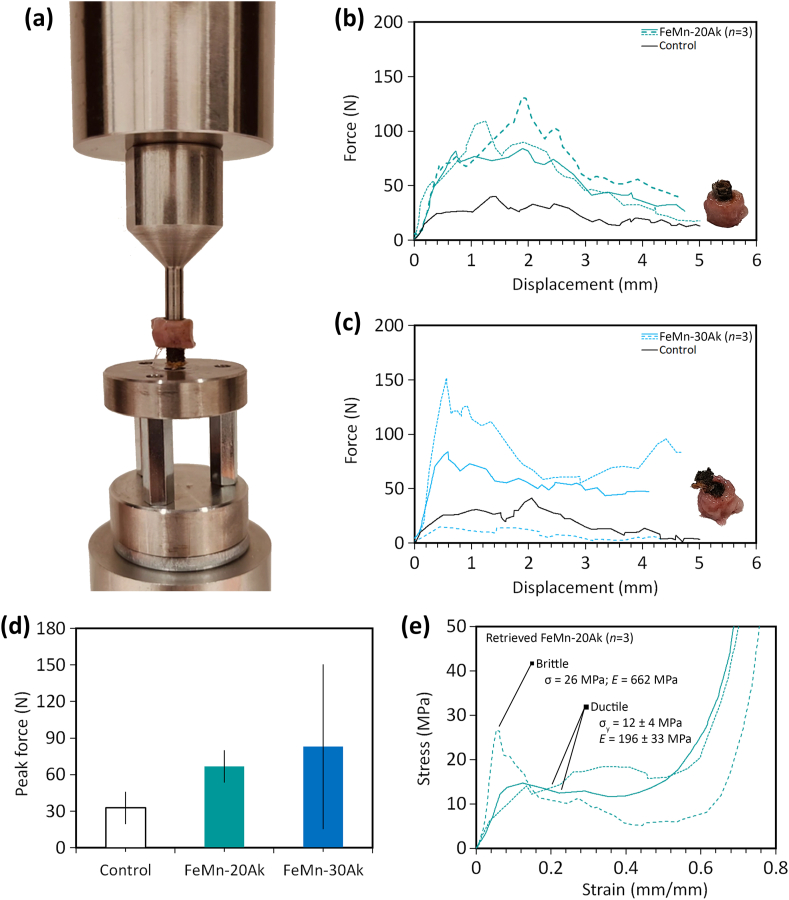
*Ex vivo* mechanical properties of the FeMn-Ak scaffolds. (a) Mechanical push-out test setup with the scaffold pushed out of the bone construct. The force and displacement curves measured during the mechanical push-out tests of (b) FeMn-20Ak (*n* = 3) and (c) FeMn-30Ak specimens (*n* = 3). (d) The peak force values were determined from the push-out test's force and displacement curves. (e) The uniaxial compressive stress-strain curves of the retrieved FeMn-20Ak specimens after 16 weeks *in vivo* (*n* = 3).

The longitudinal μCT images (Fig. 4) showed that the scaffolds were in close contact with the surrounding bone ring. Over time, we observed a closer integration of bone tissue towards the implants. Moreover, we evaluated osseointegration by measuring the forces required to push out the scaffolds from the bone rings. Overall, the peak forces needed to push out the FeMn-20Ak scaffolds (Fig. 7b) and FeMn-30Ak scaffolds (Fig. 7c) after 16 weeks *in vivo* were higher than those required for the day 0 specimens (Fig. 7d). The retrieved FeMn-20Ak scaffolds appeared intact after the push-out tests, with shear strength values (*i.e.*, 11 ± 2 MPa) comparable to the initial yield strength of the scaffolds (*i.e.*, 11 ± 2 MPa). This suggests that the higher push-out forces than those of the day 0 specimens were primarily a result of osseointegration. On the other hand, the FeMn-30Ak scaffolds were no longer intact after the push-out tests. The shear strength values for pushing out the FeMn-30Ak scaffolds (*i.e.*, 12 ± 9 MPa) exceeded their initial yield strength (*i.e.*, 1.2 ± 0.3 MPa). This indicates that the measured push-out forces resulted from osseointegration and the scaffold's resistance to plastic deformation during the test.

**Fig. 8:**
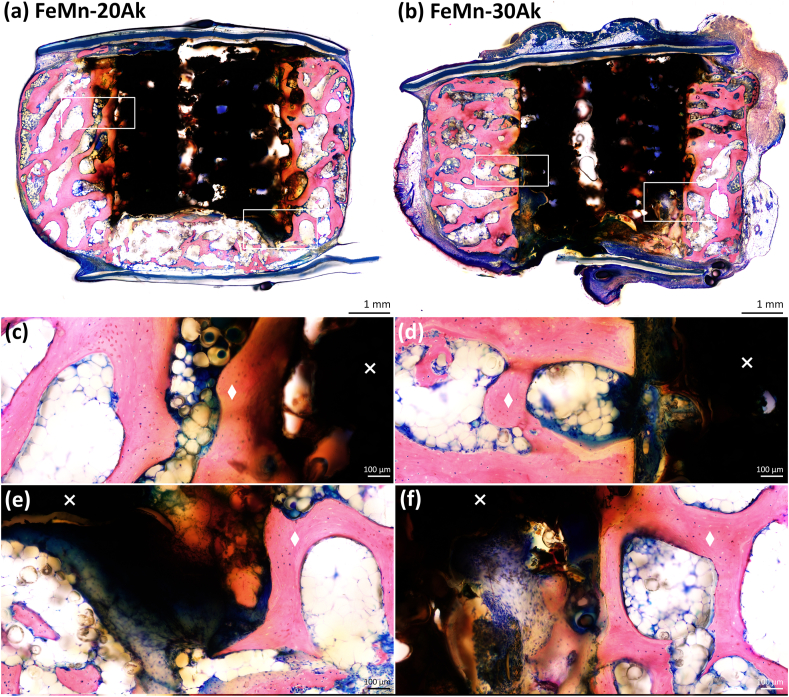
*In vivo* osseointegration capacity of the FeMn-akermanite scaffolds. Histological images at low magnification showing good integration of the (a) FeMn-20Ak and (b) FeMn-30Ak scaffolds with the surrounding bone tissue after 16 weeks *in vivo* (*n* = 3). Histological images at higher magnification showing the interface of the (c, e) FeMn-20Ak and (d, f) FeMn-30Ak scaffolds with bone tissue. The cross signs indicate the scaffold location and the diamond signs indicate the bone tissue.

The preserved structural integrity of the retrieved AM porous FeMn-20Ak scaffolds enabled us to perform compressive tests to evaluate the *in vivo* mechanical properties of the scaffolds (Fig. 7e). Under uniaxial compression, one retrieved FeMn-20Ak scaffold exhibited a brittle behavior with a compressive strength of 26 MPa and an elastic modulus of 662 MPa. However, the other two retrieved FeMn-20Ak scaffolds exhibited a ductile behavior with an average yield strength value of 12 ± 4 MPa and elastic modulus value of 196 ± 33 MPa. These values were slightly higher than the scaffolds' initial yield strength (*i.e.*, 11 ± 2 MPa) and elastic modulus (*i.e.*, 174 ± 32 MPa), suggesting that the scaffolds could maintain their mechanical integrity even after 16 weeks *in vivo*. The FeMn-30Ak scaffolds had lost their structural integrity after the push-out test and could not be evaluated.

The longitudinal μCT images (Fig. 4) demonstrated the osseointegration potential of the porous FeMn-Ak scaffolds. At the end of the *in vivo* tests, higher resolution μCT images of the constructs showed a clear presence of newly formed tissue in the critical-size defects, particularly visible on top and/or bottom of the scaffold since the scaffold did not fill the entire length of the defect (Fig. 4). The 3D reconstructed μCT images confirmed that the scaffolds in both groups were well integrated with the surrounding bone. They even had the tissue grown into them, clearly showcasing the tissue repair capacity of these scaffolds (Fig. 4c and d). Quantitative measurements showed that the BV/TV values (*i.e.*, total newly formed tissue in the critical-size defect) were comparable for both groups (*i.e.*, 17 ± 5 %, Fig. 4e). The FeMn-30Ak scaffolds contained a larger volume of newly formed tissue in the macro-pores of the scaffolds (*i.e.*, 44 ± 9 %, *p* < 0.001) compared to the FeMn-20Ak scaffolds (*i.e.*, 27 ± 5 %, Fig. 4e), suggesting there was more bone regeneration in the group with a higher volume fraction of Ak.

We closely examined the potential of the FeMn-Ak scaffolds for osseointegration. On the transversal cross-section, the FeMn-20Ak and FeMn-30Ak scaffolds were in extremely close contact with the surrounding bone ring (Fig. 5, Fig. 6a). Macro-pores (Fig. 5b–d) and struts (Fig. 6b–d) of the FeMn-Ak scaffolds were infiltrated by a newly formed tissue-like structure, which was revealed by EDS mapping to have high concentrations of C and O elements. Traces of Ca and Si elements stayed confined with the C and O elements, suggesting that the biodegradation products of Ak might have participated in the tissue regeneration process. Higher magnification images indicated that both FeMn-Ak scaffold types were integrated with the surrounding bone tissue (Fig. 5, Fig. 6e). EDS line analysis of the chemical compositions across the scaffold-to-bone interfaces indicated the new tissue formation, as evidenced by the higher C content (*i.e.*, at 60–80 μm in Fig. 5f and at 190–210 μm in Fig. 6f along the EDS line analysis). The longitudinal cross-sections of the constructs revealed similar features on the struts and macro-pores of the FeMn-20Ak and FeMn-30Ak scaffolds (Figs. S4c and S5c). The SEM-EDS analysis suggested that the FeMn-Ak scaffolds facilitated tissue regeneration on their porous surfaces. Furthermore, the histological images confirmed the occurrence of osseointegration in both scaffold groups with no clear differences (Fig. 8a and b). The defects underneath the scaffolds were filled with newly formed bone tissue, fatty tissue, and cells. Clearly, ingrowth of bone occurred within the pores of the scaffolds along the length of the interior of the bone/scaffold interface (Fig. 8e and f).

## 4 Discussion

In this study, we have performed the first *in vivo* evaluation of the extrusion-based AM porous FeMn-Ak composite scaffolds using a murine semi-orthotopic subcutaneous bone defect model. The scaffolds exhibited *in vivo* biodegradation, with 6–8 % volume losses after 16 weeks of implantation. *In vivo* excellent osseointegration was confirmed by μCT, histological analysis, EDS, and mechanical push-out tests. New bone tissue formed adjacent to the scaffolds and started infiltrating the macro-pores at the periphery of the porous scaffolds, as evidenced by SEM, μCT, and histological analyses. Our results demonstrated that the AM porous FeMn-Ak composite scaffolds showed osseointegration in critical-size bone defects, signifying the potential applications of such biomaterial in bone defect repair.

Our results showcase the potential to advance AM porous Fe-based scaffolds as biodegradable bone substitutes. It is generally accepted that biodegradable bone substitutes should have a biodegradation rate of 0.2–0.5 mm/year [36,37] that matches the bone regeneration rate, depending on the anatomical location of the bone defects. The AM porous FeMn-Ak scaffolds demonstrated a steady biodegradation profile *in vitro* [24], with 27–31 % volume losses (or 0.24–0.27 mm/year) that aligns with the expected rates. However, *in vivo* tests revealed slower biodegradation, with 6–8 % volume losses after 16 weeks. The slower *in vivo* biodegradation rates observed in this *in vivo* study are comparable to the values reported in the literature. One study reported the *in vivo* biodegradation rate of the Fe35Mn alloy to be 0.065 mm/year after 12 weeks [13]. Another study reported a 10–21 % volume reduction of the Fe30Mn alloy after 48 weeks of implantation [27]. The slow *in vivo* biodegradation of the Fe-based scaffolds is believed to be due to the formation of dense oxide layers of degradation products on the scaffold surface that hinder the transport of oxygen, which is essential for the biodegradation [38]. Based on linear regression of the observed *in vivo* biodegradation rates, we estimate that the complete biodegradation of the AM porous FeMn-Ak scaffolds would take much longer than the typically expected 1–2 years for biodegradable implants [39]. This extended timeframe may open a new prospect for Fe-based scaffolds in orthopedic applications requiring prolonged structural support during bone tissue repair.

Biodegradable bone implants must maintain their mechanical properties high enough for 12–24 weeks to provide mechanical support during bone healing [39]. Here, we observed that the porous FeMn-20Ak scaffolds maintained structural integrity over 16 weeks *in vivo*, whereas the FeMn-30Ak scaffolds were brittle. The reduced mechanical integrity of the FeMn-30Ak composite scaffolds, compared to that of the FeMn-20Ak scaffolds, is mainly attributed to the higher volume fraction of reinforcing bioceramic particles, which leads to an enhanced embrittlement effect of the ceramic phase on the metal matrix [[40], [41], [42]] in combination with the high degradation rate of the former [24]. The differences between both scaffold types are consistent with the findings of our previous *in vitro* evaluation, where FeMn-20Ak scaffolds maintained their ductility after 28 days *in vitro* biodegradation, while the FeMn-30Ak scaffolds exhibited brittle characteristics [24]. Moreover, after 16 weeks *in vivo*, the elastic modulus and yield strength of the FeMn-20Ak scaffolds were preserved and remained in a similar range to those of the human trabecular bone [43]. While further studies with larger sample sizes are needed, these preliminary findings highlight the mechanical resilience of AM porous biodegradable Fe-based composite scaffolds, particularly the FeMn-20Ak group, supporting the next research step – repairing defects in weight-bearing bones of large animals.

The discrepancy between *in vitro* and *in vivo* mechanical properties, particularly observed on the FeMn-30Ak scaffolds, was due to the dynamic biological environment encountered *in vivo*, which included fluid flow, cell activity, protein interactions, pH fluctuations, and inflammatory responses. To narrow the gaps between the *in vitro* and *in vivo* testing conditions, attempts have been made to modify *in vitro* biodegradation testing setups by introducing fluid flow to mimic physiological fluid movement in bone [44], including proteins [45,46], applying cyclic loading [47,48], or simulating inflammatory conditions during bone healing [49]. However, to the best knowledge of the authors, there is no report on *in vitro* immersion tests combining all the relevant physiological parameters simultaneously.

The *in vivo* osseointegration of the AM porous FeMn-Ak scaffolds in critical-sized bone defects, as evidenced by SEM, μCT, and histological images, correlates with the *in vitro* Ca/P forming ability on the FeMn-Ak scaffold surface. The tight bonding between FeMn-Ak scaffolds and the surrounding bone tissue highlights the advantages of using Fe-bioceramic composites compared to other biodegradable metals, such as Mg-based biomaterials, which release hydrogen gas and may impair osseointegration [50]. Our *in vivo* osseointegration results are consistent with the observations on Fe-CaP composites [51]. In addition, new bony ingrowth was integrated into the pores of the Fe0.6P alloy after one year of implantation in the femur of rabbits [14]. While our *in vivo* results are consistent with the findings of other *in vivo* studies on AM porous Fe-based scaffolds (*i.e.*, Fe-CaSiO_3_ composite, Fe30Mn alloy, and Fe35Mn alloy [[25], [26], [27]]), we report the first *in vivo* evaluation of multi-material Fe-based composite scaffolds comprising Fe, Mn, and Ak fabricated *in situ* using extrusion-based AM technique.

In the present study, we aimed to translate the development of the extrusion-based AM porous FeMn-Ak composite scaffolds from *in vitro* [24] to *in vivo*. Biomaterials for critical-size bone defect repair are typically studied in large animals, such as goats or sheep [52], due to their bone macrostructure being similar to human bones, their body weights being similar to those of adult humans, and the large availability of bone volume for implantation. However, due to the costs associated with large animal models, smaller animal models are often the first choice for initial *in vivo* biocompatibility screening of newly developed biomaterials. A few AM porous biodegradable FeMn scaffolds have been evaluated in rat and rabbit models [26,27]. Unlike those *in vivo* studies, we carried out the *in vivo* evaluation of the AM biodegradable porous FeMn-Ak composite scaffolds in a mouse semi-orthotopic subcutaneous bone defect model. This model enables the testing of bone with a defect volume one order of magnitude greater than what is currently possible in rodents [29]. This reduces the number of animals required, the overall costs for *in vivo* experiments, and the impacts on the animal.

While this model provides a practical and reproducible platform for the preliminary evaluation of the biocompatibility and osseointegration potential of the scaffolds, it presents several limitations in accurately mimicking human bone repair. The subcutaneous environment lacks the mechanical loading conditions inherent to orthotopic skeletal sites. Additionally, species-specific differences in bone metabolism, immune response, and healing dynamics between mice, bovine and humans may influence the translational relevance of the findings. These limitations underscore the need for future studies in larger load-bearing orthotopic models to more closely replicate the clinical scenarios.

Our results indicated the biosafety of the AM porous FeMn-Ak scaffolds for the vital organs. Although mild signs of hemosiderosis and manganism were observed in the liver, there was no evidence of hepatic damage. These morphological changes are likely a consequence of the high implant-to-body-weight ratio in the mouse model and are not anticipated to occur in clinical scenarios. When translated to a larger animal or human patient – where typically a single implant would be used – the relative exposure of biodegradable implant would be much lower.

Overall, the development of AM porous biodegradable Fe-based scaffolds for bone substitution has reached a remarkable milestone, showcasing several key advancements. Notably, alloying Fe with Mn has eliminated its ferromagnetic nature, while reinforcing the scaffolds with the bioactive ceramic has accelerated bone tissue regeneration [[53], [54], [55]]. We fabricated porous Fe-based scaffolds using an extrusion-based AM technique, with 35 wt% Mn and 20 or 30 vol% Ak incorporated. Previous *in vitro* evaluation confirmed that both composite scaffolds exhibited a paramagnetic behavior, being comparable to the clinically used Ti6Al4V alloy [24]. This magnetic response is primarily attributed to the complete diffusion of Mn into Fe, forming the γ-FeMn phase in the composite scaffolds during sintering at 1200 °C for 6 h [24]. Here, we demonstrated that the *in vivo* biodegradation rate of AM porous FeMn-Ak scaffolds was tunable depending on the volume fraction of Ak components. Both biodegradable FeMn-Ak scaffold groups, with 20 and 30 vol% Ak, demonstrated osseointegration in critical-sized defects. Among them, the FeMn-20Ak scaffolds stand out in terms of superior mechanical integrity. Our results from the initial evaluations using a small animal model suggest that the biodegradable AM porous FeMn-20Ak scaffolds are ready to advance to the next step of preclinical evaluation. Future studies should employ a larger orthotopic animal model in a fully immune-competent animal and incorporate detailed histopathological analyses, including quantification of immune cells, vascular density, and fibrotic response, to better understand the host response to the FeMn-Ak scaffolds.

## 5 Conclusions

In this study, we evaluated the performance of the extrusion-based AM biodegradable porous FeMn-Ak composite scaffolds for the first time for critical-size bone defect repair. These porous FeMn-Ak composite scaffolds exhibited cytocompatibility and mechanical properties in the range of the human trabecular bone. Moreover, the scaffolds could attract Ca/P deposition during *in vitro* biodegradation and enable osseointegration in critical-size bone defects. Our study highlights the potential and challenges of the porous FeMn-akermanite composite scaffolds as bone substitutes, encouraging further research in large animal models.

## CRediT authorship contribution statement

**Niko E. Putra:** Writing – review & editing, Writing – original draft, Visualization, Methodology, Investigation, Formal analysis, Conceptualization. **Jietao Xu:** Writing – review & editing, Writing – original draft, Methodology, Investigation, Formal analysis, Conceptualization. **Marius A. Leeflang:** Methodology, Investigation. **Nicole Kops:** Methodology, Investigation. **Maria Klimopoulou:** Methodology, Investigation. **Vahid Moosabeiki:** Software, Investigation. **Lidy E. Fratila-Apachitei:** Writing – review & editing, Resources, Methodology. **Jie Zhou:** Writing – review & editing, Supervision, Resources, Methodology, Conceptualization. **Gerjo J.V.M. van Osch:** Writing – review & editing, Supervision, Resources, Methodology, Conceptualization. **Eric Farrell:** Writing – review & editing, Supervision, Resources, Methodology, Conceptualization. **Amir A. Zadpoor:** Writing – review & editing, Supervision, Resources, Conceptualization.

## Generative AI in scientific writing

The authors did not use AI or AI-assisted technologies in the writing process.

## Declaration of competing interest

The authors declare that they have no known competing financial interests or personal relationships that could have appeared to influence the work reported in this paper.

## Data Availability

Data will be made available on request.
