# Supplementary material for: Additively manufactured biodegradable porous FeMn-akermanite scaffolds for critical-size bone defects: the first in vivo evaluation
Source: Mater Today Bio. 2025 Jul 21;34:102123. doi: 10.1016/j.mtbio.2025.102123 (PMC12308005; doi:10.1016/j.mtbio.2025.102123)
Supplement: Multimedia component 1 [file mmc1.docx]

**Supplementary materials**


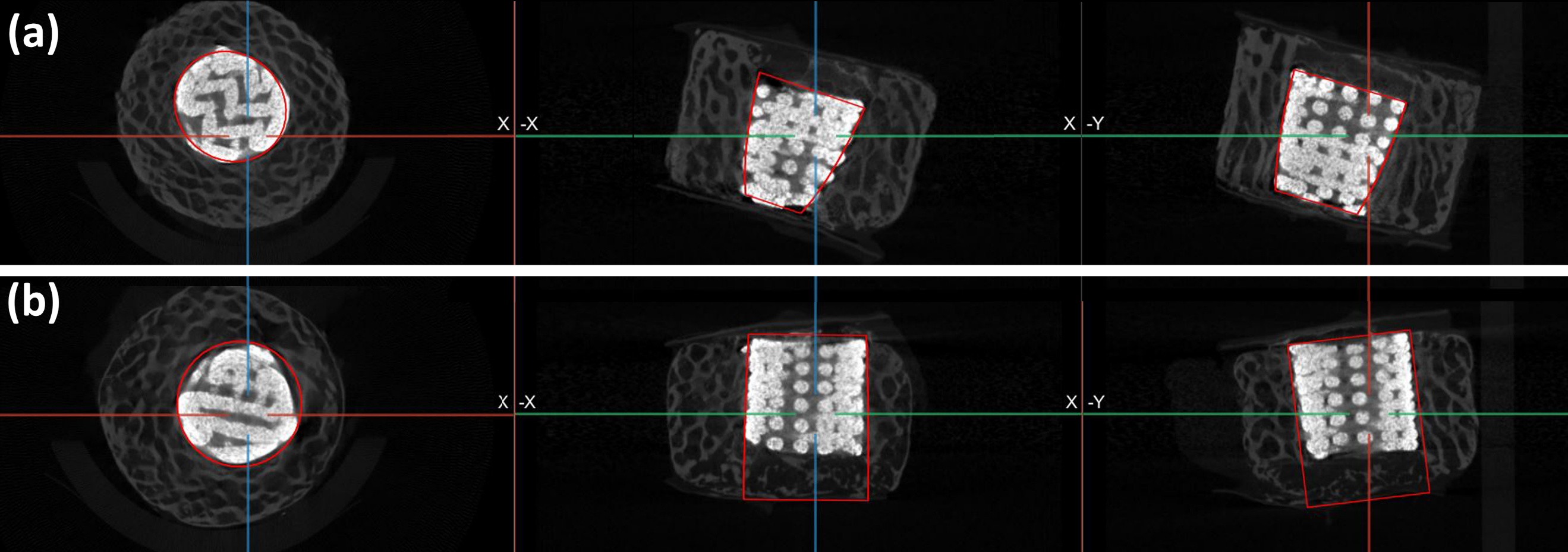


**Figure S1**. Representative images indicating the selection of the ROI of (a) scaffold and (b) the total defect.


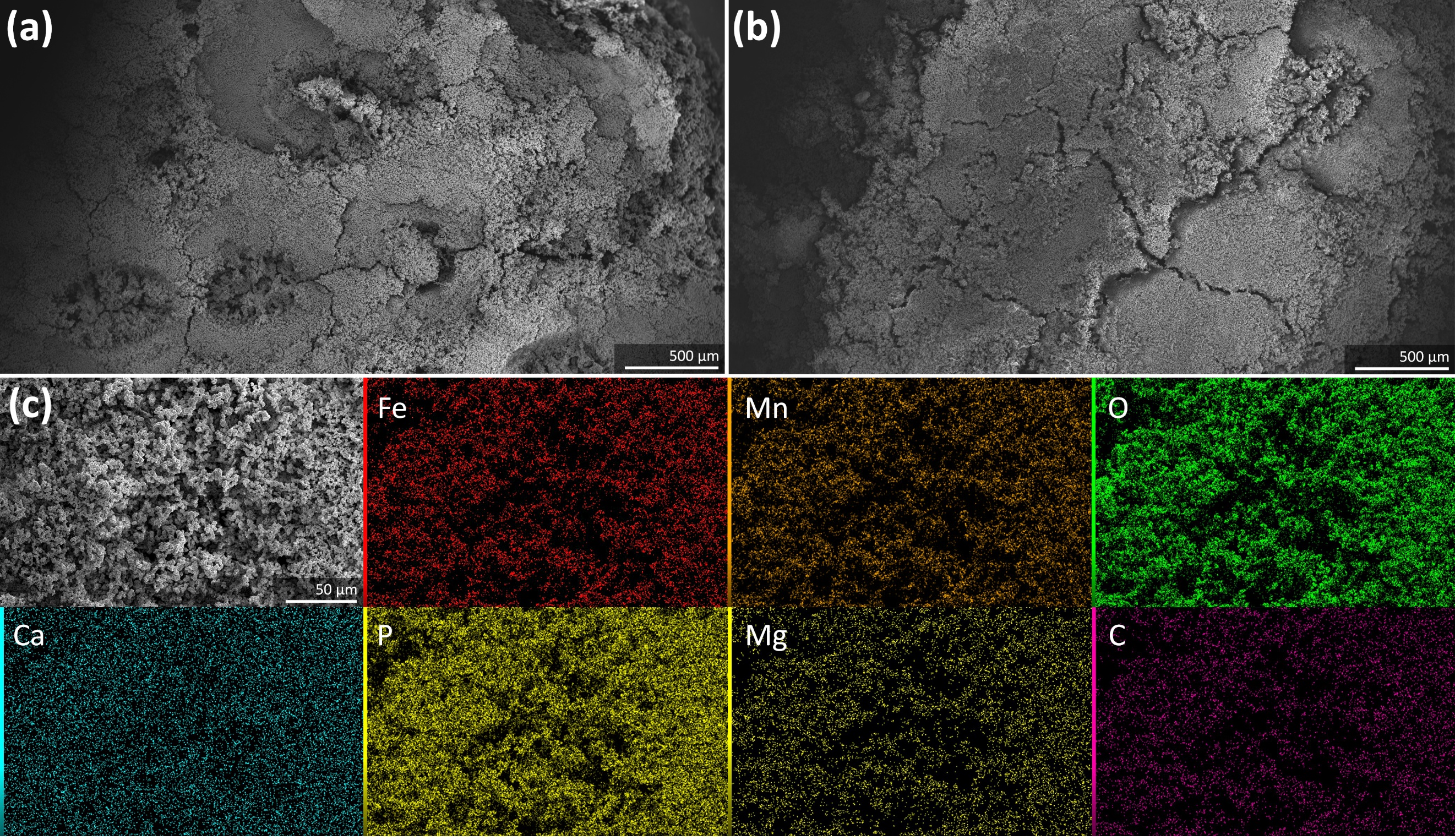


**Figure S2.** The SEM image of biodegradation products on the surface of (a) FeMn-20Ak and (b) FeMn-30Ak. (c) SEM image and EDS elemental mapping of biodegradation products on the surface of the FeMn-30Ak scaffolds after 8 weeks in the r-SBF medium.


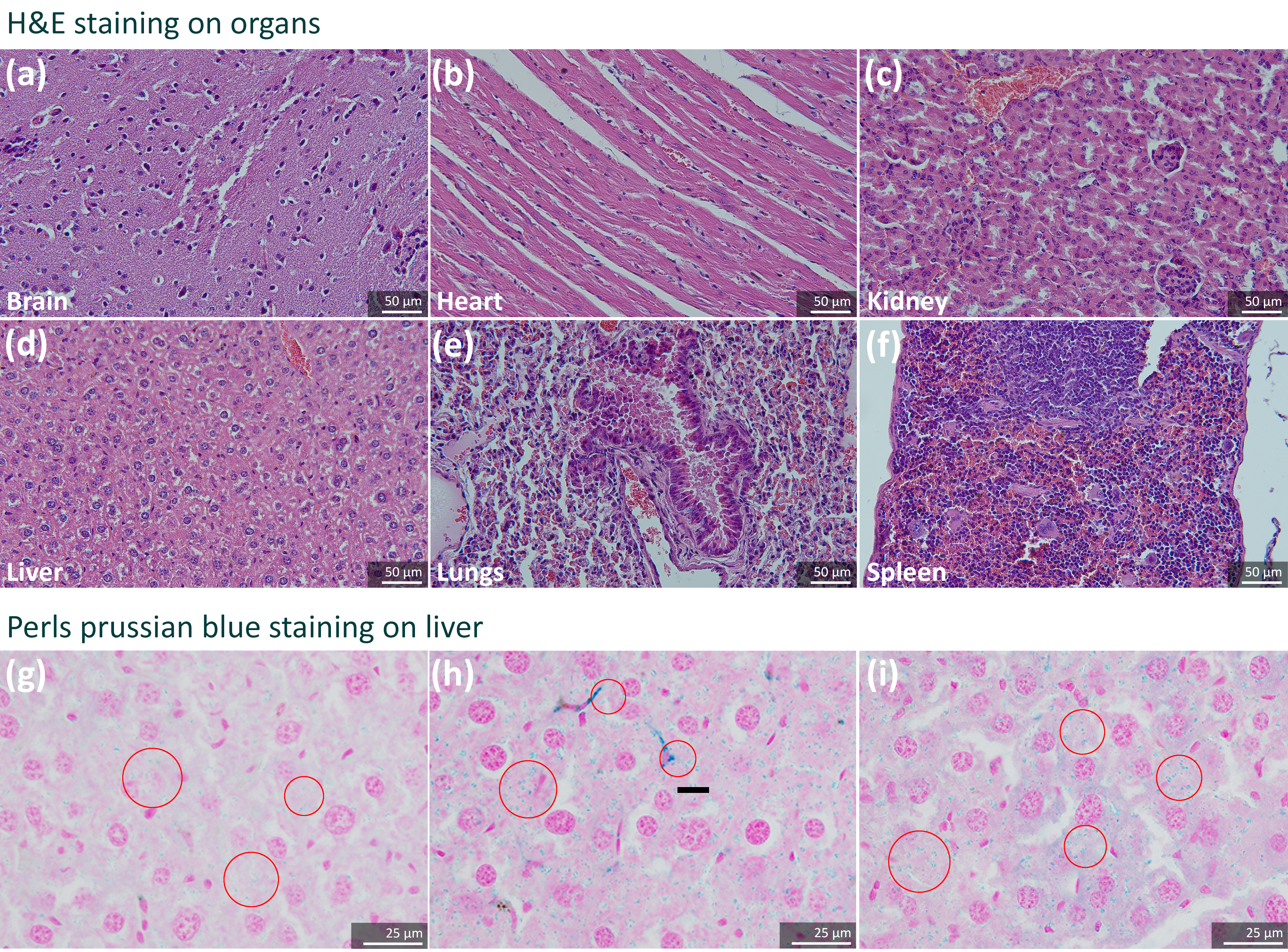


**Figure S3.** Microscopic images of the H&E-stained sections of organs: (a) brain, (b) heart, (c) kidney, (d) liver, (e) lungs, and (f) spleen. Microscopic images of the Perls Prussian blue stained sections of liver with (g) mild and (h, i) excess accumulation of iron or manganese in Kupffer cells (as pointed by the red circles), indicating hemosiderosis or manganism, without hepatic damage.

**
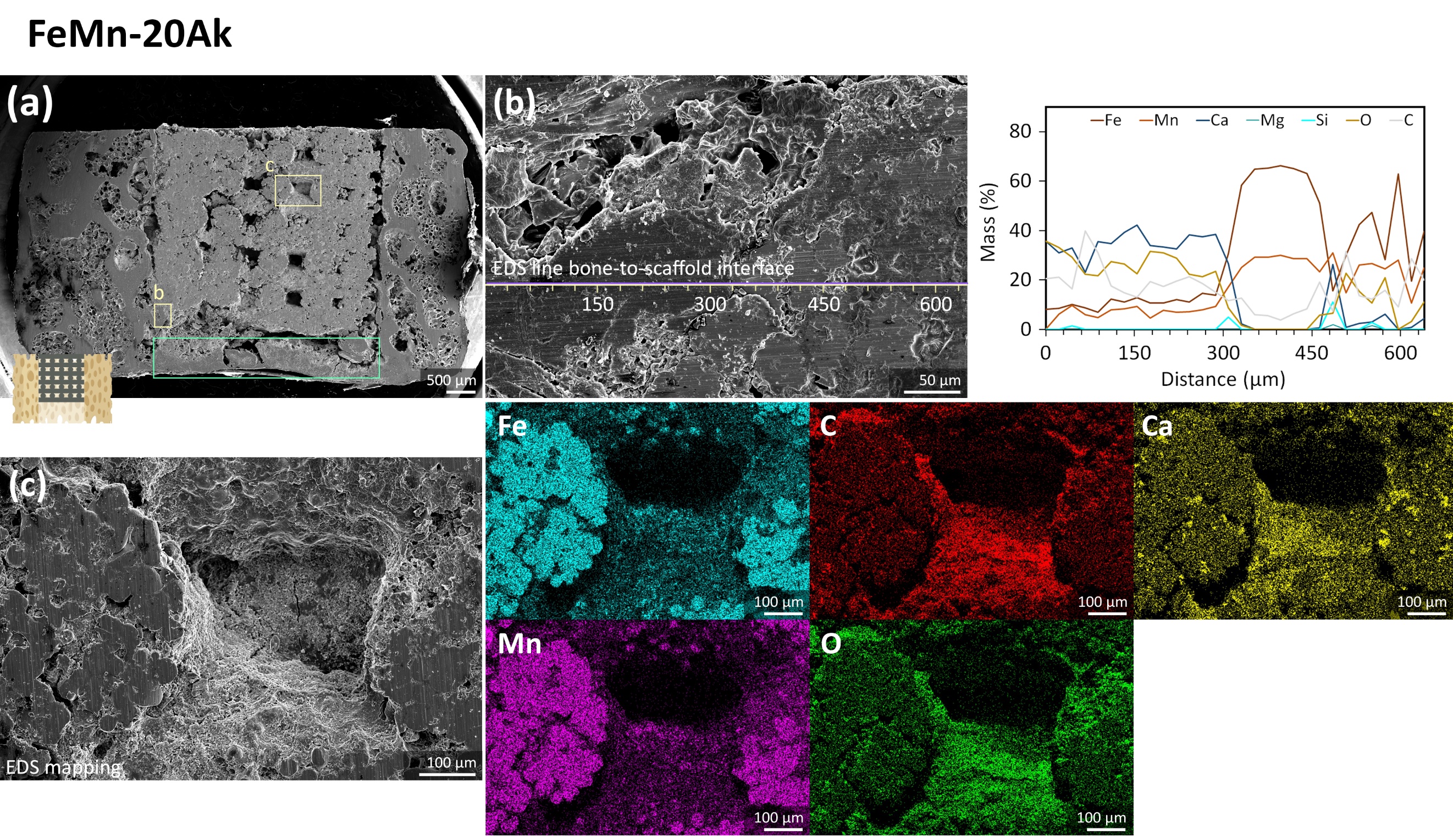
**

**Figure S4**. (a) The longitudinal cross section of the FeMn-20Ak bone-scaffold construct, showing tissue regeneration beneath the scaffold (green rectangle). (b) EDS line analysis across the bone-to-scaffold interface. (c) EDS elemental mapping at the strut and macro-pore site.


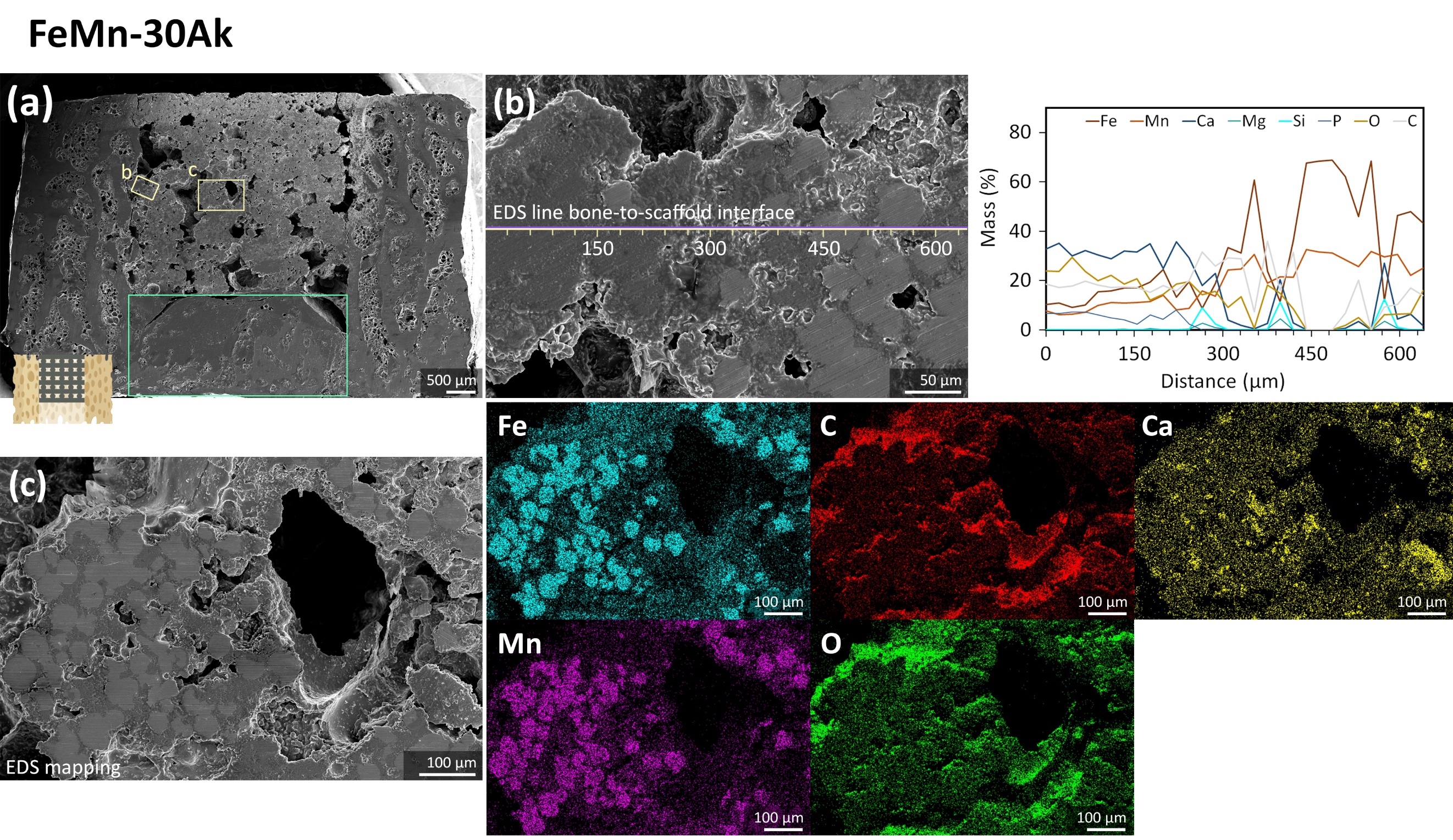


**Figure S5**. (a) The longitudinal cross section of the FeMn-30Ak bone-scaffold construct, showing tissue regeneration beneath the scaffold (green rectangle). (b) EDS line analysis across the bone-to-scaffold interface. (c) EDS elemental mapping at the strut and macro-pore site.

**Table S1.** Ion concentrations of the total human blood plasma and the r-SBF

| **Ion** | **Concentration (mM)** | |
| --- | --- | --- |
|  | **in total human blood plasma** | **in r-SBF** |
| **Na^+^** | 142.0 | 142.0 |
| **K^+^** | 5.0 | 5.0 |
| **Mg^2+^** | 1.5 | 1.5 |
| **Ca^2+^** | 2.5 | 2.5 |
| **Cl^-^** | 103.0 | 103.0 |
| **HCO_3_^-^** | 27.0 | 27.0 |
| **HPO_4_^2-^** | 1.0 | 1.0 |
| **SO_4_^2-^** | 0.5 | 0.5 |
